# The Role of Cerebrolysin in Promoting Axonal Regeneration and Functional Recovery after Peripheral Nerve Injury: A Focus on Macrophage Activation

**DOI:** 10.34172/apb.025.46084

**Published:** 2025-10-11

**Authors:** Aida Karimian, Arash Abdolmaleki, Asadollah Asadi, Saber Zahri, Hussein A. Ghanimi

**Affiliations:** ^1^Department of Biology, Faculty of Science, University of Mohaghegh Ardabili, Ardabil, Iran; ^2^Department of Biophysics, Faculty of Advanced Technologies, University of Mohaghegh Ardabili, Namin, Iran; ^3^College of Nursing, University of Al-Ameed, Karbala, Iraq

**Keywords:** Neuroprotective, Cerebrolysin, Macrophage, Nerve regeneration

## Abstract

**Purpose:**

The research investigated the neuroprotective properties of Cerebrolysin regarding functional recovery, axonal regeneration, and macrophage polarization in a rat model of acute sciatic nerve damage.

**Methods:**

The research included 72 male Wistar rats, categorized into six groups: sham control, crush injury, vehicle-treated crush damage, and two groups receiving Cerebrolysin treatment for crush injury. The assessment of functional recovery was conducted with the sciatic functional index and hot plate test, while axonal regeneration, muscle atrophy, and macrophage polarization were also studied.

**Results:**

Results demonstrated that Cerebrolysin, particularly at 5 mg/kg, significantly improved SFI scores and thermal paw withdrawal latency compared to the control group, indicating enhanced functional recovery. Histomorphometric analysis revealed increased myelinated axon counts in the Cerebrolysin-treated groups. Cerebrolysin also reduced gastrocnemius muscle atrophy and induced a change in macrophage polarization from pro-inflammatory M1 to pro-healing M2.

**Conclusion:**

These results imply that cerebrolysin improves functional outcomes, promotes axonal regeneration, and modifies macrophage polarization to provide neuroprotective effects in peripheral nerve damage. The 5 mg/kg dosage proved to be more effective than the 2.5 mg/kg dose. This study highlights the potential of Cerebrolysin as a therapeutic agent for peripheral nerve injuries.

## Introduction

 Peripheral nerve injuries (PNIs) affect more than 90,000 individuals annually, representing a significant global health concern. In the United States alone, the associated loss of sensory and motor function contributes to an estimated $150 billion in annual healthcare costs. Current therapeutic strategies primarily involve surgical repair and the administration of anti-inflammatory agents aimed at improving recovery and achieving sustained functional outcomes.^1^ Compared to the central nervous system, peripheral nerve axons have a greater capacity for regeneration; however, this depends on a number of variables, including the kind of damage, the patient’s age, the distance from the lesion site, and the time between the injury and the therapeutic intervention.^2^ According to experimental data, distal injuries result in a more favorable recovery since the axon distance to the target tissue is shorter, and crushed nerves mend more readily than transected ones.^3^ Clinical data demonstrates that severe PNIs, resulting in amputation or nerve tissue excision, often lead to sensory and motor impairments in the absence of appropriate therapeutic intervention, underscoring the need for the optimization of treatment strategies for nervous system injuries.^4^

 For nerve regeneration, axons must possess the capacity for regrowth, the environment of the distal segment must facilitate the development of repairing axons, and the target tissues must be amenable to reinnervation.^2^ Damaged neurons’ gene transcription is impacted by retrograde impulses from injury sites, resulting in a repair phenotype. More than 1000 genes have been altered in dorsal root ganglion neurons, according to microarray study; transcription factors that encode these genes are essential.^5^

 After peripheral nerve injury, Schwann cells (SCs) and neurons undergo regenerative adaptations. The repair of the distal nerve segment involves multiple sequential stages, including Wallerian degeneration, axonal regrowth, and reinnervation of the target organ.^6^ Although Wallerian degeneration promotes neural regeneration, immune cell infiltration and SC transformation are necessary for effective repair. In this process, macrophages are essential because they mediate the breakdown of distal axons, remove debris, release neurotrophic factors, control the production of cytokines, and rebuild the extracellular matrix. These functions establish macrophages as key contributors to Wallerian degeneration.^7^ As phagocytic elements of the innate immune system, macrophages either infiltrate or live within injured neurons and are essential for peripheral nerve regeneration. Efficient clearance of cellular debris is critical, since residual material not only obstructs but also generates inhibitory signals for regenerating axons. Furthermore, hypoxic circumstances resulting from peripheral nerve damage might activate macrophages to enhance angiogenesis and release vascular endothelial growth factor (VEGF) in the distal nerve segment.^8^ Macrophages have emerged as therapeutic targets in PNI owing to their pivotal functions in clearing cellular debris and secreting cytokines essential for nerve repair. The peripheral nervous system’s ability to regenerate neurons depends on the activation of macrophages, which include both the pro-inflammatory M1 and anti-inflammatory M2 phenotypes.^9^ Pro-inflammatory cytokines released by M1 macrophages encourage stem cell transdifferentiation toward a regenerative state. After that, these cells develop into M2 macrophages, which release cytokines that reduce inflammation and promote angiogenesis and nerve healing. For nerve regeneration to be successful, M1 macrophages must be polarized toward the M2 phenotype. The M2 subtypes—M2a, M2b, M2c, and M2d—promote angiogenesis, inflammation resolution, cell maturation, and proliferation. These subtypes show promise as targets for treatment.^10^ Regenerating axons must arrive to the target muscle within a crucial window of time for reinnervation to be effective since extended delays impair muscle tissue’s receptivity. SCs preserve the basal lamina tubes, which serve as guiding structures for axonal regrowth following injury.^11^ Chronic denervation leads to SC atrophy, resulting in the degeneration and loss of the basal lamina and Büngner bands. Consequently, muscle tissue undergoes atrophy, with alterations in fiber size and number. These changes highlight the importance of timely and effective therapeutic intervention, particularly in cases of severe peripheral nerve injury.^12^

 Numerous techniques have been suggested for the healing of PNIs, including stem cell transplantation, neurotrophic factor administration, axonal sprouting enhancement, myelination augmentation, muscle atrophy prevention, and optimized organ reinnervation, to restore lost cells.^13^ Mitigating the propagation of secondary injuries resulting from trauma can serve as an efficacious intervention for PNIs. Traumatic injuries to the nervous system are classified into primary and secondary injuries. Primary injuries manifest immediately following nerve damage, resulting in mechanical harm and neuronal cell death. Secondary injuries encompass pathological alterations that exacerbate tissue damage over time. Factors contributing to secondary injuries in the nervous system include inflammation, reduced blood flow, hypoxia, elevated free radicals, intracellular calcium ion influx, activation of proteolytic enzymes, and excitotoxicity due to glutamate release.^14,15^ In this sense, administering neuroprotective substances is a viable method of reducing secondary harm and neuronal death brought on by peripheral nerve injury.^16^ Because it can efficiently pass the blood–brain barrier, Cerebrolysin, a neuroprotective and neurotrophic substance, is used to treat brain lesions.

 Neuropeptides and growth factors from pig brain, such as brain-derived neurotrophic factor (BDNF), nerve growth factor (NGF), and glial cell line-derived neurotrophic factor (GDNF), make up this neuroprotective drug. These substances directly affect neuronal cells.^17^ In Eastern Europe, Russia, and Asia, Cerebrolysin is often given to treat Parkinson’s disease, epilepsy, strokes, dementia, chronic cerebrovascular illnesses, and brain injuries.^18,19^ Moreover, certain research outcomes indicate that this medication improves cognitive abilities in individuals diagnosed with attention deficit hyperactivity disorder and autism.^20^ Mostly via improving neuronal metabolism and stopping the spread of secondary damage and cell death, Cerebrolysin exerts protective properties on the central nervous system.^21^

 Cerebrolysin’s neuroprotective benefits in the central nervous system have been the subject of much investigation, but its impact in PNIs has received very less attention. Using male Wistar rats in a model of traumatic sciatic nerve damage, the current work attempts to evaluate the neuroprotective potential of Cerebrolysin.

## Materials and Methods

###  Animals and ethics statement

 The research involved 48 adult male Wistar rats with weights ranging from 200 to 250 g. The animals were maintained in Plexiglas cages under controlled laboratory conditions, with unrestricted access to food and water. Environmental conditions were maintained at 22 ± 2 °C, 60 ± 5% relative humidity, and a 12-hour light/dark cycle. All experimental procedures adhered to the ARRIVE guidelines and complied with U.K. regulations for animal research.

 The Ethics Committee of Mohaghegh Ardabili University (Iran) accepted all procedures, which were carried out in accordance with the Animals (Scientific Procedures) Act of 1986 and its related standards, as well as the EU Directive 2010/63/EU on animal experimentation.

###  Surgical method

 Peripheral nerve damage was induced in this investigation using the sciatic nerve crush (CRUSH) model, and all surgical operations were carried out aseptically. Ketamine (60 mg/kg) and xylazine (10 mg/kg) were injected intraperitoneally to induce anesthesia. Rats were put on a heating pad that was kept at 37 ± 1 °C while they were in the prone posture. Using forceps with a 5 mm tip, the left sciatic nerve was crushed 15 mm proximal to the trifurcation for 30 seconds after a longitudinal incision was created from the greater trochanter to the mid-thigh. Closure was accomplished by using 4-0 nylon sutures to close the skin and 6-0 VICRYL absorbable sutures to close the layers of muscle and fascia. The animals were observed until they had fully regained awareness. After surgery, a bitter-tasting nail polish was put under the operated ankle to avoid autotomy, and a single subcutaneous dosage of buprenorphine (0.05 mg/kg) was given for analgesia. The sham group had the same surgical exposure, but the sciatic nerve was left unharmed. The muscle and skin were then sutured.^22^

###  Experimental groups and animal treatment

 Two experimental groups were given intraperitoneal injections of Cerebrolysin (EVER Pharma, A-4866) at doses of 2.5 mg/kg or 5 mg/kg body weight, respectively, after sciatic nerve crush. A total of 48 rats were randomly assigned into four groups (n = 12 per group): (i) a sham control group, in which the sciatic nerve was exposed without crush injury; (ii) a negative control group that underwent sciatic nerve crush without treatment.

 The first week after traumatic peripheral nerve damage is crucial for the onset of subsequent injuries; hence, this trial included a 7-day medication therapy regimen commencing immediately post-surgery.^9^ The pharmacological dosages administered were determined based on findings from prior trials.^23^

###  Sciatic functional index (SFI)

 To gauge the recovery of motor function, the SFI was measured before surgery and subsequently every week for eight weeks. Rats were given black ink on their hind limbs and let to go down a hallway lined with paper in order to analyze their footprints. A blinded researcher measured the experimental (E) and contralateral normal (N) limbs’ paw length (PL; distance from the tip of the third toe to the heel), toe spread (TS; distance between the first and fifth toes), and intermediary toe spread (ITS; distance between the second and fourth toes). Each hind limb had three measurements, which were then averaged. Next, the SFI was determined using the formula Bain et al. (1989) described: [(EPL − NPL)/NPL] − 38.3 [SFI] + 109.5 [(ETS − NTS)/NTS] + 13.3 [(EIT − NIT)/NIT] − 8.8.^24^ The range of SFI readings is 0 (normal motor function) to -100 (total loss of hindlimb motor function).^25^

###  Hot plate test

 The hot plate test, which measures the operated limb’s thermal paw withdrawal reflex latency as a measure of nociceptive threshold, was used to evaluate sensory function. The delay between paw contact with the surface and withdrawal was measured while the rats were on a heated plate (PE34, IITC Life Sciences, USA) that was kept at 55 ± 1 °C. The mean value was determined after each test was run three times at two-minute intervals. A 12-second cut-off period was used to avoid tissue damage.^26^

###  Histomorphometry analysis

 After the studies, animals were killed and sciatic nerve segments were removed 10 mm from the crush site. After fixing overnight in 2.5% glutaraldehyde at 4 °C, samples were dehydrated in graded ethanol and embedded. Transverse slices (1 µm thick) were stained with toluidine blue (Carl Zeiss, Germany) for light microscopy. ImageJ (National Institutes of Health, Maryland, USA) was used to quantify axon number, diameter, myelin thickness, fiber diameter, and G-ratio by a blinded researcher. As a structural biomarker of optimum myelination, the G-ratio (axonal diameter/total fiber diameter) was utilized. Sciatic nerve samples from the undamaged contralateral limb were healthy controls.^27^

###  Gastrocnemius muscle mass ratio

 Assessing nerve reinnervation and muscle atrophy using the gastrocnemius muscle mass ratio. The investigation ended with gastrocnemius muscle excision from both hind limbs and wet weight recording. The ratio was computed by dividing the operated (left) limb’s muscle weight by the contralateral unaffected (right) limb. Values around one suggested less muscle atrophy and improved reinnervation.^28,29^

###  Masson trichrome staining

 Masson’s trichrome staining was used to measure muscle atrophy in the operated limb. Graded ethanol concentrations were used to dehydrate the excised gastrocnemius muscles after they had been fixed in paraformaldehyde. Following xylene clearing, tissues were fixed in paraffin and longitudinally sectioned at a 5 µm thickness. After being stained with Masson’s trichrome, the sections were seen using a light microscope (Carl Zeiss, Germany). Image-Pro Plus 6.0 software was used to obtain digital pictures and quantify the cross-sectional areas of muscle fibers. The healthy control was the contralateral, undamaged muscle.^30,31^

###  Immunohistochemical staining

 Two weeks post-surgery, immunohistochemical staining was performed to quantify macrophage populations in injured sciatic nerves. Four animals per group were perfused intracardially with cold phosphate-buffered saline (PBS), followed by 4% paraformaldehyde (PFA) in PBS. Sciatic nerves, 10 mm distal to the compression site, were harvested and post-fixed in 4% PFA for 24 hours, then cryo-embedded in Tissue Tek O.C.T. compound for an additional 24 hours. Frozen sections (12 µm thickness) were cut and blocked with 10% fetal bovine serum (FBS) in PBS for 1 hour at room temperature. Sections were incubated overnight at 4°C with primary antibodies: mouse anti-rat CD68 (1:100, IgG1; Bio-Rad, MCA341R) to label total macrophages, CD86 (1:100, IgG1; Bio-Rad, MCA2874) to detect M1 macrophages, and CD206 (1:100, IgG; Santa Cruz Biotechnology) to identify M2a/M2c macrophages. Following three washes with PBS containing 0.1% Triton X-100, sections were incubated for 1 hour at room temperature with secondary antibodies diluted in PBS with 0.1% Triton X-100: Alexa Fluor 488 (green)-conjugated goat anti-mouse IgG1 (1:100; Bio-Rad, STAR132) for CD68, Alexa Fluor 594 (red)-conjugated goat anti-mouse IgG1 (1:100; Bio-Rad, STAR132) for CD86 detection, and Alexa Fluor 647 (magenta)-conjugated goat anti-mouse IgG (1:100; Invitrogen) for CD206 detection. Finally, stained sections were visualized using a Zeiss Axioskop 2 fluorescence microscope with FITC, Cy3, and Cy5 filters.^32,33^

###  Enzyme-linked immunosorbent Assay (ELISA)

 Seven days post-injury, during the peak of inflammation, ELISA was used to quantify pro-inflammatory cytokines (IL-1β and IL-6), anti-inflammatory cytokines (IL-4, IL-10, and TGF-β), and growth factors (NGF and VEGF) in regenerated nerve tissue, following manufacturer protocols. Nerve samples (5 mm proximal and distal stumps) were homogenized in protease inhibitor-containing RIPA buffer and centrifuged at 12,000 × g for 20 minutes at 4 °C. Cytokine concentrations in supernatants were measured using commercial ELISA kits for Rat IL-1β (Invitrogen, BMS630), Rat IL-4 (Sigma-Aldrich, RAB0302), Rat IL-6 (ERA32RB), and Rat IL-10 (A78898). At 450 nm, optical density (OD) was measured and compared to a seven-point reference curve in pg/mg tissue.

###  Statistical analysis

 SPSS software version 25.0 (IBM Corp., Chicago, IL, USA) was employed to conduct statistical analyses. The Kolmogorov-Smirnov test was employed to assess the normality of the data. The non-parametric Kruskal-Wallis and Mann-Whitney tests were employed to conduct group comparisons for non-normally distributed data, while the one-way analysis of variance (ANOVA) followed by Tukey’s post hoc test was employed to analyze normally distributed data. Quantitative data was represented as the mean ± standard deviation (SD), while descriptive results were presented as percentages. Statistical significance was defined as a *P* value of less than 0.05.

## Results

###  SFI

**Figure 1:**
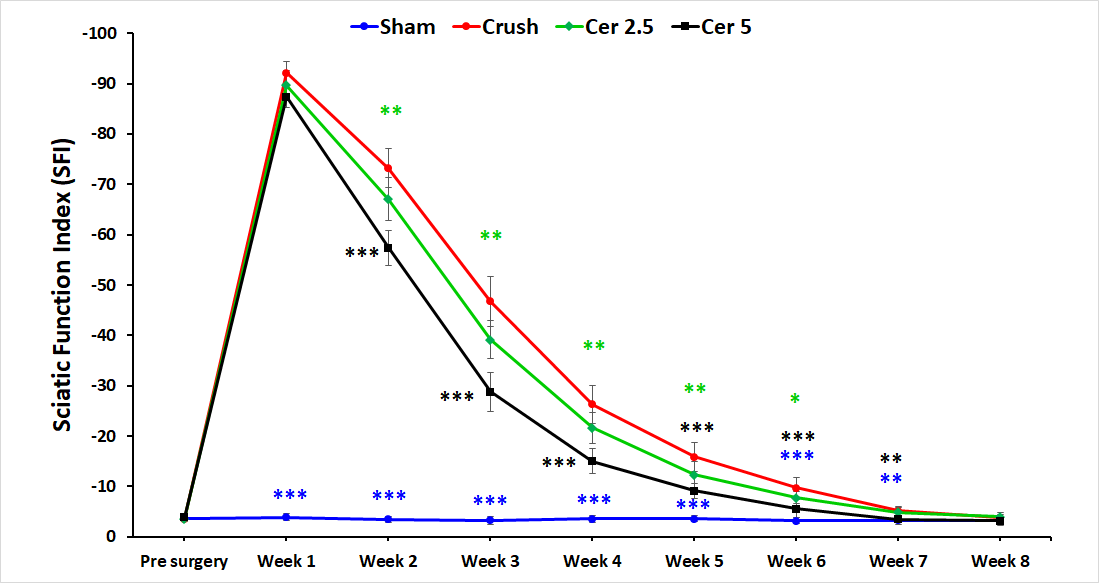
Motor recovery assessment using SFI ratings following sciatic nerve injury in rats. SFI scores are presented pre-surgery and at weekly intervals post-injury for the sham surgery group and groups treated with different doses of Cerebrolysin (2.5 mg/kg and 5 mg/kg). All groups exhibited complete paralysis at Week 1, with SFI values approaching -100. Notably, the 5 mg/kg Cerebrolysin group demonstrated significantly improved motor recovery compared to other groups at Weeks 1, 3, and 5 (*P* < 0.05). While both Cerebrolysin groups showed recovery by Week 8, the 5 mg/kg group maintained superior performance during the critical intermediate recovery phase (Weeks 4-7). The sham surgery group showed no significant effect on SFI scores. The data is presented as mean ± standard deviation (SD), and statistical significance is denoted as * *P* < 0.05, * *P* < 0.01, and *** *P* < 0.001 for comparison with crush group

 Weekly SFI ratings were used to assess motor recovery after sciatic nerve damage; 0 denoted normal function and 100 denoted total motor disability. Before surgery, all groups demonstrated normal motor performance and baseline SFI values (Figure 1). One-week post-injury, rats in all nerve injury groups showed complete paralysis, with mean SFI scores approaching −100. Over the subsequent weeks, SFI values gradually improved across all groups. Notably, rats administered 5 mg/kg Cerebrolysin demonstrated significantly enhanced motor recovery compared to other injury groups at Weeks 1, 3, and 5 (*P*< 0.05). While rats receiving 2.5 mg/kg Cerebrolysin showed moderate functional improvement during the early recovery phase (Weeks 2–3), those treated with 5 mg/kg Cerebrolysin exhibited a more pronounced and accelerated recovery from Week 4 onward, with SFI values consistently nearing 0. This superior performance persisted through the intermediate phase (Weeks 4–7), highlighting the greater efficacy of the higher Cerebrolysin dose in promoting motor function recovery. By Week 8, SFI values for both Cerebrolysin groups converged, indicating similar final outcomes; however, the 5 mg/kg group maintained a clear advantage during the critical intermediate period. Sham surgery had no effect on SFI scores.

###  Sensory recovery evaluation

**Figure 2:**
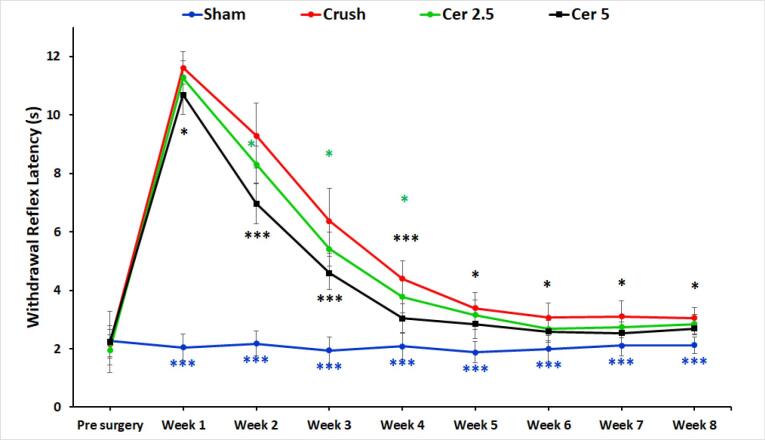
Sensory recovery assessment through withdrawal responses to thermal stimulation in the hot plate test. Baseline latencies were approximately 2 seconds for all groups. Following injury, the Crush, 2.5 mg/kg, and Cer 5 mg/kg groups showed significant increases in latency, peaking at 10–12 seconds. The Sham group maintained latencies near baseline. Over 8 weeks, withdrawal latencies decreased in the injured groups, with the Cer 5 group demonstrating the fastest recovery, particularly during Weeks 3 to 5. By Weeks 6–8, both Cerebrolysin groups neared baseline values, while the Crush group lagged behind. These findings highlight the superior efficacy of 5 mg/kg Cerebrolysin in promoting sensory nerve recovery. The data is presented as mean ± standard deviation (SD) (n = 10), and statistical significance is denoted as * *P* < 0.05 and *** *P* < 0.001 for comparison with crush group

 Sensory function recovery was evaluated based on withdrawal responses to thermal stimulation. Figure 2 shows hot plate test results for the four groups preoperatively and weekly up to eight weeks post-surgery. Baseline withdrawal reflex latencies were consistent across all groups at approximately 2 seconds, indicating normal function. Following injury, the Crush, Cer 2.5, and Cer 5 groups exhibited a significant increase in latency, peaking around 10–12 seconds, reflecting substantial nerve impairment and delayed reflexes. The Sham group-maintained latency near baseline, indicating minimal or no damage. Over time, withdrawal latencies in the Crush, Cer 2.5, and Cer 5 groups progressively decreased, signifying gradual recovery of nerve function. When compared to the Crush and Cer 2.5 groups, the Cer 5 group showed the quickest and most significant recovery, especially during the intermediate period (weeks 3–5), as shown by considerably lower latency values (*P* < 0.05). During the initial phase (Weeks 1–2), recovery patterns were similar between the Cer 5, Cer 2.5, and Crush groups, with no significant differences observed. However, from Week 3 onward, the Cer 5 group exhibited accelerated recovery, outperforming the other groups. By Weeks 6–8, Cer 5 and Cer 2.5 latency discrepancies had decreased, with both reaching baseline values but remained somewhat higher than the Sham group. These data show that 5 mg/kg Cerebrolysin promotes sensory nerve regeneration during the intermediate period better than 2.5 mg/kg and the untreated Crush group. By Week 8, both Cer 5 and Cer 2.5 groups exhibited near-complete recovery, while the Crush group lagged behind. The Sham group remained unaffected throughout, serving as a stable control. Statistical significance observed at multiple time points underscores the reliability of these results.

###  Histomorphometry analysis

**Figure 3:**
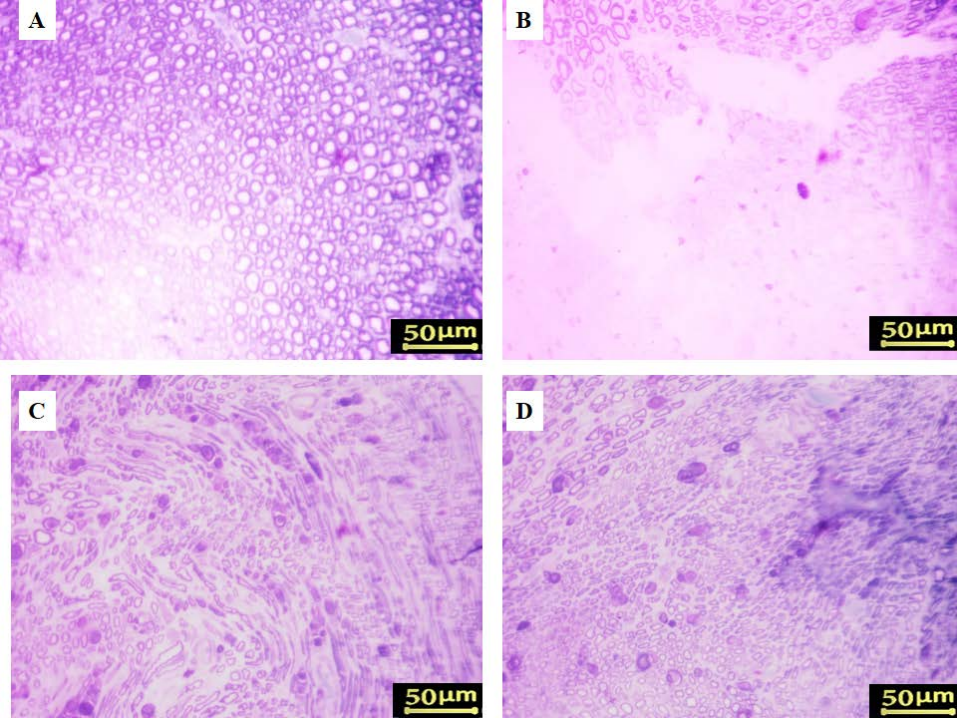
Toluidine blue staining of regenerated nerves. (A) Sham group, (B) Crush group, (C) Cerebrolysin 2.5 mg/kg, and (D) Cerebrolysin 5 mg/kg

 Axonal regrowth was assessed by histomorphometric examination of sciatic nerves 5–8 mm distal to the coaptation site. Toluidine blue staining showed diminished myelin sheaths and axonal diameters in the control group compared to the sham group (Figure 3). Morphometric analysis showed that all sciatic nerve crush animals had greater G-ratios, denser fiber distribution, smaller axonal diameters, lower myelin thickness, and more myelinated axons than sham rats (Table 1). Animals administered with 5 mg/kg Cerebrolysin had significantly more myelinated axons than those receiving 2.5 mg/kg *(P* < 0.001; Table 1). Neither Cerebrolysin group had significant variations in axonal diameter, myelin thickness, or G-ratio. Additionally, the 2.5 mg/kg Cerebrolysin group showed substantial improvements in all morphometric parameters compared to the untreated control group (*P* < 0.001).

**Table 1:** Histomorphometric assessment of myelinated axons in cross-sections of regenerated sciatic nerves 8 weeks post-surgery

	**Myelinated fiber count**	**Myelinated fiber diameter (µm)**	**Axon diameter (µm)**	**Myelin sheath thickness (µm)**	**G-ratio**
Sham	7669 ± 219***	6.73 ± 0.31***	4.34 ± 0.30***	1.19 ± 0.04***	0.64***
Crush	8536 ± 283	3.23 ± 0.49	1.93 ± 0.29	0.65 ± 0.10	0.57
Cer (2.5 mg/kg)	8961 ± 287*	3.99 ± 0.5*	2.38 ± 0.28*	0.81 ± 0.11*	0.59
Cer (5 mg/kg)	9747 ± 562***	5.59 ± 0.64***	3.61 ± 0.59***	0.99 ± 0.10***	0.63**

*Note*. Results are presented as mean ± SD. * *P* < 0.05, ** *P* < 0.01, and *** *P* < 0.001 indicate significance compared to the crush group.

###  Gastrocnemius muscle atrophy

**Figure 4:**
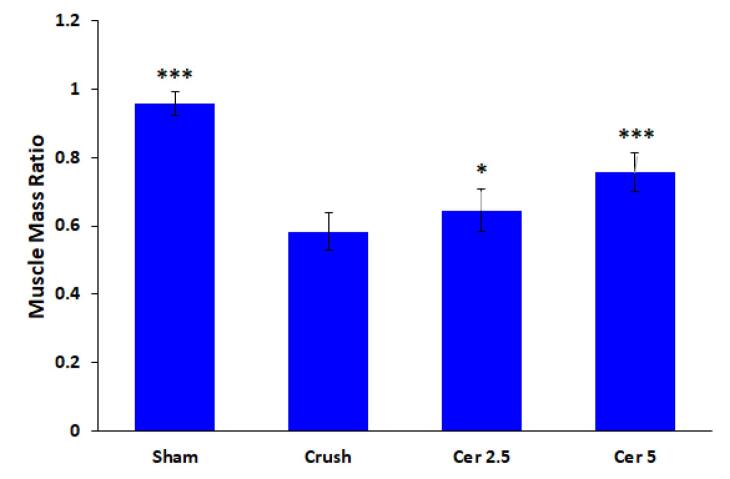
Evaluation of gastrocnemius atrophy using muscle mass ratio, 8 weeks post-surgery. The muscle mass ratio is expressed as a percentage of the undamaged contralateral side. The results show that the Sham group (A) exhibited normal muscle mass, while the Crush group (B) displayed significant gastrocnemius muscle atrophy and fiber degeneration. Treatment with Cerebrolysin at doses of 2.5 mg/kg (C) and 5 mg/kg (D) significantly reduced muscle atrophy and improved muscle mass ratios compared to the control group, with the 5 mg/kg Cerebrolysin group demonstrating the greatest improvement. The data is presented as mean ± standard deviation (SD) (n = 10). Statistical significance is denoted as * *P* < 0.05 and *** *P* < 0.001 for comparison with crush group

 Masson’s trichrome staining and the muscle mass ratio were used to measure gastrocnemius atrophy eight weeks after surgery. The proportion of the contralateral side that was undamaged was used to represent the muscle mass ratio. The findings demonstrated that, in comparison to the sham group, all rats who had their sciatic nerve crushed (Figure 4) had gastrocnemius atrophy and fiber degradation. Compared to the control group, treatment with Cerebrolysin at 2.5 mg/kg and 5 mg/kg (Figure 4D) considerably decreased muscle atrophy and increased the muscle mass ratio; the most noticeable benefit was obtained with the 5 mg/kg dosage (*P* < 0.001).

**Figure 5:**
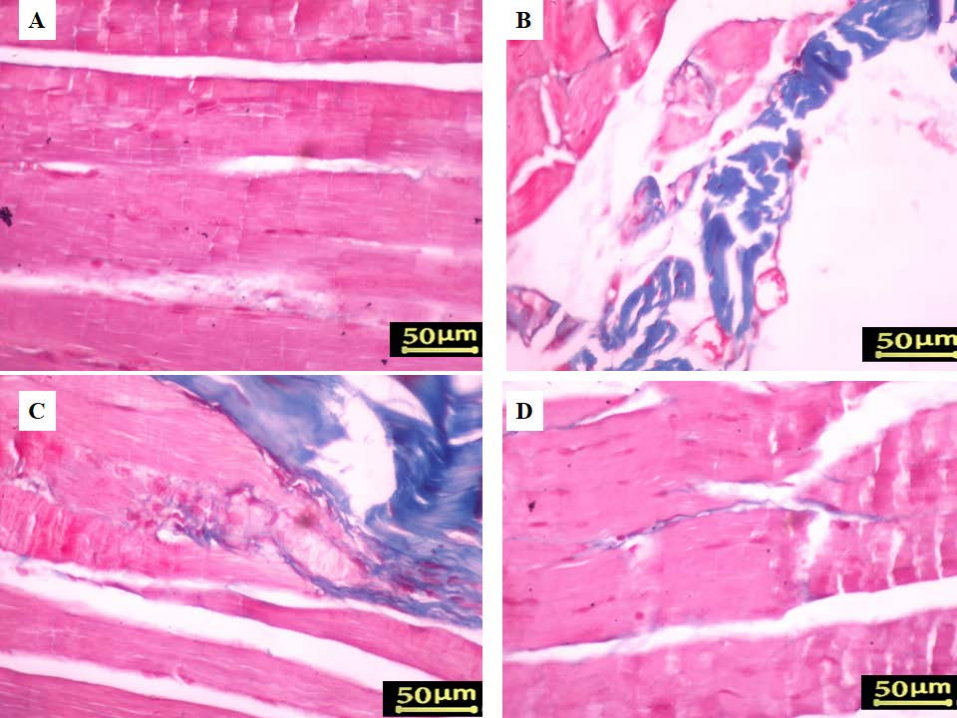
Assessment of gastrocnemius muscle histology using Masson’s trichrome staining, 8 weeks post-surgery. The Sham group (A) displayed a typical gastrocnemius muscle with minimal fibrous connective fibers. In contrast, the Crush group (B) showed denervated muscles characterized by increased fibrous connective tissue and heightened muscle fiber breakdown. Treatment with Cerebrolysin significantly improved muscle atrophy compared to the control group. Notably, rats receiving 5 mg/kg of Cerebrolysin (C) exhibited reduced muscular atrophy and less fibrous connective tissue compared to those treated with 2.5 mg/kg of Cerebrolysin (D)

 Additionally, denervated muscles in rats with sciatic nerve injury showed substantial connective tissue deposition and enhanced muscle fiber degeneration (Figure 5B), whereas the gastrocnemius muscle of the sham group showed negligible fibrous connective tissue according to Masson’s trichrome staining (Figure 5A). During the 8 weeks post-surgery, Cerebrolysin therapy reduced gastrocnemius muscle atrophy relative to the control group. The findings indicated that rats administered 5 mg/kg of Cerebrolysin (Figure 5C) had less muscular atrophy and fibrous connective tissue compared to those receiving 2.5 mg/kg of Cerebrolysin (Figure 5D).

###  The ratio of M1 to M2 macrophages in sciatic nerve

 Cerebrolysin’s effects on macrophage polarization were examined by immunostaining nerve tissues for CD68, CD86, and CD206 two weeks after surgery.

**Figure 6:**
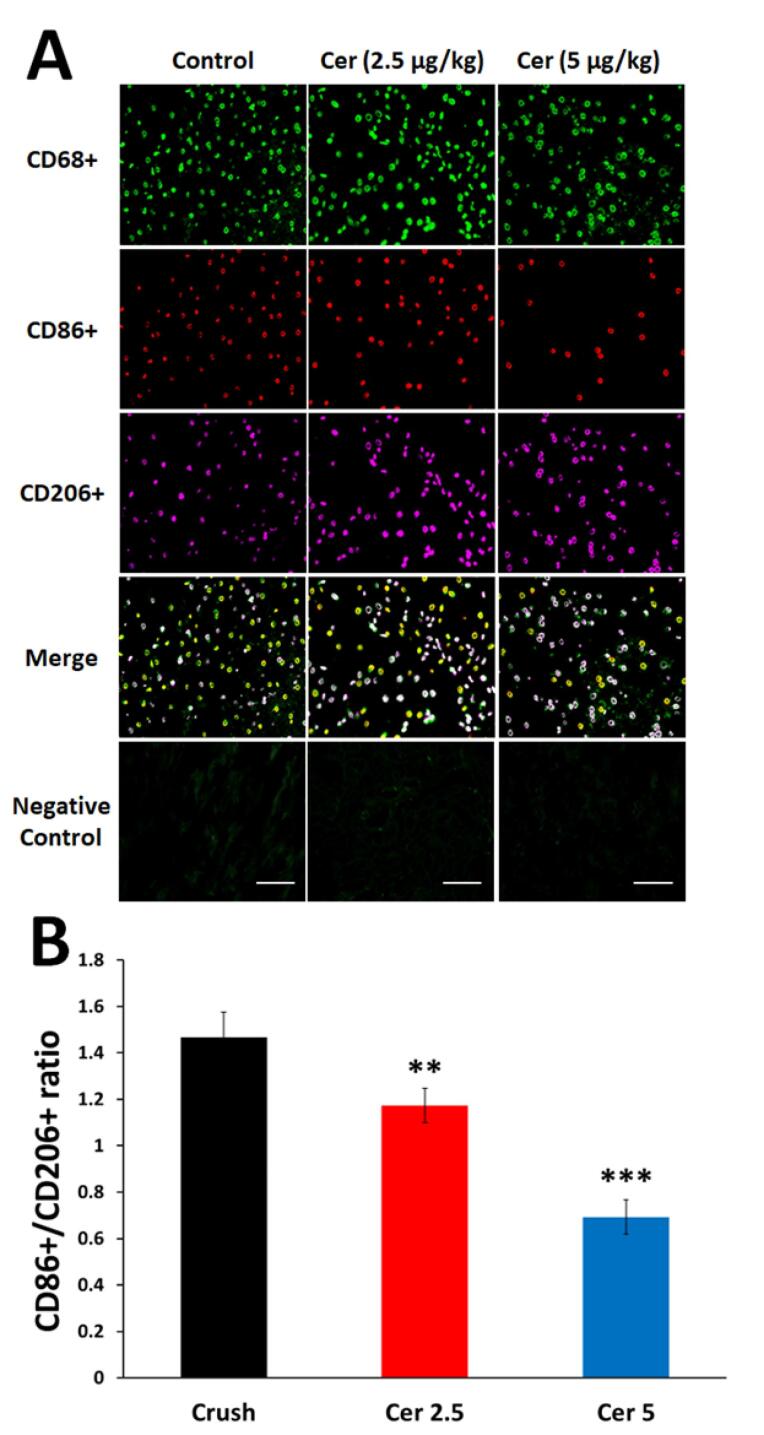
Impact of Cerebrolysin treatment on macrophage polarization in the sciatic nerve. (A) Triple immunofluorescent staining of resident macrophages in the sciatic nerve, highlighting the M0 marker (CD68/ green), M1 marker (CD86 + /red), and M2 marker (CD206 + /magenta). (B) The ratio of pro-inflammatory macrophages (CD86 + ) to pro-healing macrophages (CD206 + ). Data are presented as mean ± SD (n = 4). ** *P* < 0.01 and *** *P* < 0.001 indicate significant differences compared to the crush group. (Scale bar 200 µm)

Figure 6 displays CD206 + (M2a and M2c) and CD86 + (M1) macrophages in all experimental groups. Although Cerebrolysin did not significantly change macrophage counts, immunohistochemical analysis showed that both dosages promoted macrophage polarization from pro-inflammatory M1 to pro-regenerative M2, resulting in a lower CD86 + /CD206 + ratio compared to controls (Figure 6; *P* < 0.01). Rats treated with 5 mg/kg Cerebrolysin showed larger effects than those given with 2.5 mg/kg (*P* < 0.001). These data show that M2 macrophages predominated over M1 macrophages in Cerebrolysin-treated rats’ damaged sciatic nerves, demonstrating that macrophage phenotype influences regeneration outcomes more than quantity.

###  Cytokine levels

**Figure 7:**
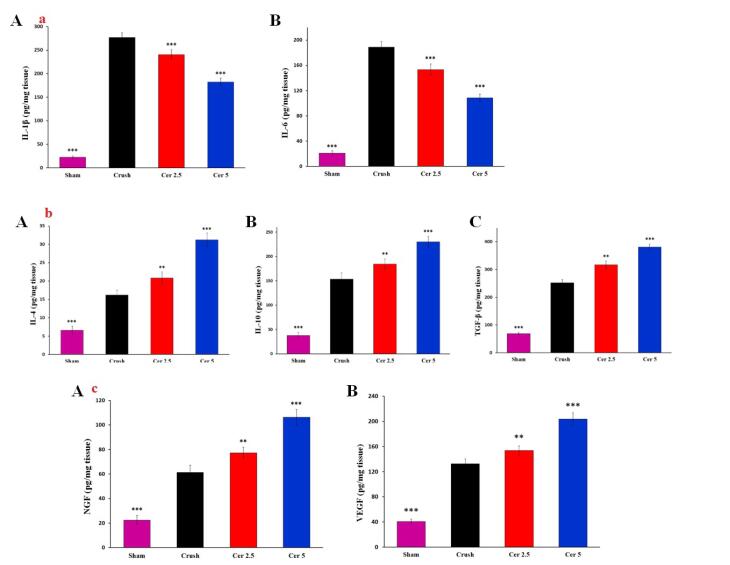
(a) ELISA results showing levels of inflammatory cytokines in the regenerating sciatic nerve of rats. The Crush group exhibited significantly higher levels of IL-1β (A) and IL-6 (B) compared to the Sham group. Treatment with Cerebrolysin (Cer 2.5 and Cer 5) significantly reduced these cytokine levels, demonstrating its strong anti-inflammatory effects. Data are presented as mean ± SD (n = 4). *** *P* < 0.001 indicate significant differences compared to the crush group. (b) ELISA results demonstrating the expression of anti-inflammatory cytokines in the regenerating sciatic nerve of rats. Cerebrolysin treatment significantly increased levels of IL-4 (A), IL-10 (B), and TGF-β (C) compared to the Crush group. Both IL-4 and IL-10 levels were markedly elevated in the Cerebrolysin-treated groups, with TGF-β showing the most significant increase, highlighting the enhanced anti-inflammatory response during nerve repair. Data are presented as mean ± SD (n = 4). ** *P* < 0.01 and *** *P* < 0.001 indicate significant differences compared to the crush group. (c) ELISA results indicating growth factor levels in the regenerating sciatic nerve of rats. The Crush group exhibited significantly elevated levels of NGF (A) and VEGF (B) compared to the Sham group. Treatment with Cerebrolysin further increased these levels, particularly in the Cer 5 group (*P* < 0.001). These findings suggest that Cerebrolysin not only reduces inflammation but also enhances nerve repair and regeneration by upregulating essential growth factors. Data are presented as mean ± SD (n = 4). ** *P* < 0.01 and *** *P* < 0.001 indicate significant differences compared to the crush group

 ELISA showed considerable cytokine and growth factor changes in regenerated sciatic nerves. The Crush group had significantly higher IL-1β and IL-6 levels than the Sham group (Figure 7 (a) A and B). In contrast, Cerebrolysin therapy (Cer 2.5 and Cer 5) dramatically lowered these levels (*P* < 0.001), indicating considerable anti-inflammatory effect.

 Administration of Cerebrolysin substantially increased the production of anti-inflammatory cytokines IL-4, IL-10, and TGF-β compared to the Crush group (Figure 7 (b) A-C). IL-4 and IL-10 levels increased significantly in Cerebrolysin-treated groups (*P* < 0.001), with TGF-β showing the greatest rise (*P* < 0.001), suggesting its involvement in encouraging nerve regeneration in an anti-inflammatory environment.

 In terms of growth factors, Crush had considerably greater NGF and VEGF concentrations than Sham. The Cer 5 group showed the greatest rise in levels after Cerebrolysin treatment (Figure 7 (c) A and B, *P* < 0.001). These findings suggest that Cerebrolysin reduces inflammation and promotes nerve repair and regeneration by upregulating growth factors.

## Discussion

 Cerebrolysin promotes functional recovery, axonal regeneration, and macrophage polarization in sciatic nerve damage, according to this research. Functional assessments via SFI and hot plate tests revealed a dose-dependent improvement, with the 5 mg/kg Cerebrolysin group showing accelerated motor and sensory recovery, particularly during the intermediate phase. These findings suggest Cerebrolysin enhances nerve regeneration through neurotrophic support, anti-inflammatory modulation, and macrophage polarization, likely involving VEGF, TGF-β, and NGF pathways. This is consistent with prior research indicating the important involvement of neurotrophic factors in axonal regeneration and remyelination.

 The findings of the SFI and hot plate tests show that Cerebrolysin improves motor and sensory recovery after sciatic nerve damage, especially when administered at a dosage of 5 mg/kg. Motor recovery was dose-dependent, with the 5 mg/kg group exhibiting accelerated improvement from Week 4 onwards, while sensory function recovery showed a similar trend, with a notable advantage during Weeks 3–5.^23^ These findings suggest that Cerebrolysin facilitates nerve regeneration through neurotrophic support, anti-inflammatory modulation, and macrophage polarization, likely involving VEGF, TGF-β, and NGF pathways.^17^ These results are consistent with previous studies demonstrating the role of neurotrophic factors in axonal regeneration, remyelination, and functional recovery after peripheral nerve damage. The findings stress Cerebrolysin’s therapeutic potential for peripheral nerve healing, highlighting the need for further research into its long-term effectiveness and underlying molecular processes. Previous studies have reported similar neuroprotective and neurotrophic benefits of Cerebrolysin in central nervous system injuries, suggesting its broader applicability in neural repair.^21^ Comparative analyses with other neurotrophic agents, such as BDNF and GDNF, may help elucidate its specific advantages in peripheral nerve regeneration.^34^ Histomorphometric evaluation demonstrated marked structural differences in axonal regeneration across the study groups, underscoring the positive impact of Cerebrolysin treatment. In the control group, degenerated myelin sheaths and reduced axonal diameters were observed, consistent with impaired regenerative capacity following sciatic nerve transection.^35^ Treatment with 5 mg/kg Cerebrolysin notably resulted in a significant increase in the number of myelinated axons compared to the 2.5 mg/kg group, highlighting a dose-dependent enhancement of axonal regeneration.^36^ However, no discernible variations in axon diameter, myelin thickness, or G-ratio were seen between the two Cerebrolysin-treated groups, indicating that although Cerebrolysin promotes axonal regeneration, its effects on myelination may level off at a certain dose.^37^ These results correspond with earlier research indicating the neuroprotective and neurotrophic properties of Cerebrolysin in nerve damage models, likely mediated via VEGF, NGF, and TGF-β pathways.^38,39^ Furthermore, the improved morphometric parameters seen in the groups treated with Cerebrolysin in comparison to the control group demonstrate the therapeutic potential of this substance in promoting neuron recovery.^40^ To determine the ideal dosage schedule for obtaining the greatest possible functional recovery and to elucidate the molecular processes by which Cerebrolysin promotes peripheral nerve regeneration, further research is necessary.^41^ As shown by an elevated CD206 + /CD68 + ratio, the study’s findings suggest that Cerebrolysin stimulates macrophage polarization from the pro-inflammatory M1 phenotype toward the pro-regenerative M2 phenotype. These findings are consistent with prior evidence demonstrating that macrophage polarization is essential for peripheral nerve regeneration, with M2 macrophages supporting repair through anti-inflammatory cytokine production, extracellular matrix remodeling, and the release of neurotrophic factors.^42^ The observed dose-dependent effect, with the 5 mg/kg Cerebrolysin group exhibiting a greater M2 response than the 2.5 mg/kg group, suggests that higher Cerebrolysin concentrations may optimize the regenerative environment by enhancing the M2-mediated repair processes.^43^ Interestingly, the total macrophage count was not significantly altered, indicating that Cerebrolysin primarily influences macrophage phenotype rather than recruitment, similar to findings in other models of neuroinflammation.^44^ These findings suggest Cerebrolysin may be an immunomodulatory agent in peripheral nerve repair by modifying the post-injury inflammatory response to promote nerve regeneration. Future studies should explore the molecular mechanisms underlying this polarization shift, particularly in relation to VEGF, TGF-β, and NGF signaling pathways, which are known to regulate macrophage phenotype transitions. The TUNEL assay results indicate that motor neuron apoptosis in the L4–L6 spinal cord follows a time-dependent pattern after sciatic nerve injury, peaking at three days and persisting for up to 21 days. This aligns with previous studies demonstrating that nerve injury triggers apoptotic pathways in spinal motor neurons due to disrupted neurotrophic support and inflammatory responses.^45^ The observed decrease in apoptosis after NGF administration points to its neuroprotective function, which is most likely mediated by SC NF-κB activation, which has been linked to enhancing axonal regeneration and neuronal survival.^46^ These results are consistent with previous research that has shown that NGF attenuates neuronal apoptosis by activating pro-survival signaling pathways, such as PI3K/Akt and MAPK/ERK, and simultaneously downregulating pro-apoptotic mediators like Bax and caspase-3.^47^ Additionally, the significant difference between the NGF-treated and untreated crush groups emphasizes NGF’s potential as a therapeutic agent for preventing secondary neuronal degeneration after peripheral nerve injury. Further investigations into the molecular mechanisms underlying NGF-mediated neuroprotection, particularly its interplay with inflammatory and apoptotic regulators, could enhance therapeutic strategies for nerve repair.

 Overall, by promoting neuroprotection, functional recovery, and immunomodulation, this work highlights Cerebrolysin’s potential as a therapeutic treatment for peripheral nerve damage. Further investigations are necessary to delineate the precise molecular mechanisms of action and to optimize dosing strategies for clinical application. Comparative studies with other neurotrophic factors, including BDNF and GDNF, may provide additional insights into its relative advantages in promoting nerve regeneration.

## Limitation

 1. We propose in future studies, it would be valuable to incorporate additional techniques, such as qPCR or Western blot, to assess CD86/CD206 expression or cytokine markers. These approaches could enhance the depth of investigation and improve the accuracy and reliability of the findings.

## Conclusion

 This study’s results indicate that one week of intraperitoneal injection of Cerebrolysin after surgical neurotomy and direct suture may improve the regeneration process and facilitate functional recovery. The neuroprotective effects of Cerebrolysin seem to be facilitated by its immunomodulatory functions and the modulation of macrophage responses to damage. However, further study is need to validate these results.

## Competing Interests

 All authors declare that they have no competing interests.

## Ethical Approval

 The Ethics Committee of Mohaghegh Ardabili University (Iran) accepted all procedures, which were carried out in accordance with the Animals (Scientific Procedures) Act of 1986 and its related standards, as well as the EU Directive 2010/63/EU on animal experimentation. Code number: IRI.22486.
